# Mental Health Professionals’ Perception of Respect for Human Rights and Organizational Well-Being in Three Countries of South America

**DOI:** 10.3390/ijerph21020214

**Published:** 2024-02-12

**Authors:** Cesar Ivan Aviles Gonzalez, Doris Marina Cerchiaro Fernandez, Martha Esther Guerra Munoz, Robert Romero Ramirez, Yessika Madelaine Abarca Arias, Maria Veronica Brasesco, Gian Mario Migliaccio, Ferdinando Romano, Giulia Cossu, Diego Primavera, Mauro Giovanni Carta

**Affiliations:** 1 Department of Nursing, Universidad Popular del Cesar, Valledupar 200001, Colombia; 2 Department of Medical Sciences and Public Health, University of Cagliari, 09042 Cagliari, Italy; 3 Faculty of Administrative, Accounting, and Economic Sciences, Rectorate and Vice-Presidency for Research, Universidad Popular del Cesar, Valledupar 200001, Colombia; 4 Academic Department of Nursing, Universidad Nacional de San Agustin de Arequipa, Arequipa 04001, Peru; 5 RIAT—Red Internacional de Acompañamiento Terapeutico, Buenos Aires B1228, Argentina; 6 Department Human Sciences and Promotion of the Quality of Life, San Raffaele Open University, 00118 Rome, Italy; 7 Department of Public Health and Infectious Diseases, Università La Sapienza, 00185 Rome, Italy

**Keywords:** human rights, organizational well-being, job satisfaction, mental health, South America, multi-site investigation

## Abstract

The respect for human rights in mental health care services significantly contributes to organizational well-being and is evolving into an actual benchmark of quality standards. This study assesses the perception of the respect for human rights for users and staff, as well as organizational and job satisfaction among mental health professionals in three South American countries, through the well-being at work and respect for human rights (WWRR) questionnaire and assesses whether there are significant differences. Seven mental health facilities in Argentina, Colombia, and Peru were involved in this observational study. The sample comprised 310 mental health professionals. The three countries exhibited differences in WWRR, particularly in the staff’s satisfaction with resources for care (η^2^ = 0.166) and staff’s satisfaction with organizational aspects (η^2^ = 0.113). Colombia had the lowest scores in these factors but the highest in the perception of the respect for human rights for users and staff, although this difference did not reach a statistical significance. Despite the progress made in recent years towards coercion-free medical standards and an increased focus on mental health polices in Latin American countries, there is a need to enhance the quality standards of mental health services, recognizing the value that the respect for human rights holds for the organizational well-being of both mental health users and professionals.

## 1. Introduction

The approval by the United Nations Assembly of the Convention on the Rights of Persons with Disabilities (CRPD) [1] has aroused a consensus in the political world and among stakeholders, which has led to the widespread adoption of the document in most countries of the world and to the ratification (therefore submitting to the control of the appropriate United Nations committee) of many states [1]. This process has fostered an enhanced consideration of the rights of people with disabilities within national policy guidelines and practices [2]. However, there is still a lack of use and improvement of these principles in social and health care practice, as demonstrated by reports of human rights violations [3,4,5].

Considering the entire spectrum of disabilities, one area that is particularly critical in terms of the respect for human rights is that of psychosocial disability [6]. This topic has been the subject of specific reports by international organizations and associations that fight for the respect of human rights [7,8,9]. The factors related to human rights violations in the mental health field include the lack of comprehensive legislation, the absence of independent legal assistance, and the lack of adequate care [10,11].

The mental health services provided are still scarce in resources, particularly in terms of human resources. Although psychosocial disability accounts for one-third of the total disability worldwide, 70% of people suffering from mental health conditions lack access to sufficient quality care [12,13]. Most people with mental health problems reside in low- and middle-income countries where, in the pre-COVID era, mental health care expenditure was less than COP 0.25/person/year [12]. Research has been found suggesting that the number of resources of mental health services influences the outcomes related to the users’ social functioning [14,15]. Numerous reports have drawn attention to the need to improve not only the number but also the quality of staff working in mental health services [16,17]; on the other hand, some studies have shown that it is possible to raise staff awareness, with excellent consequences, for respecting the users’ rights [18,19]. This point is also relevant in terms of institutional responsibilities, because according to Article 13 of the CRPD, parties must promote the training of professionals and staff working with persons with disabilities “so as to better provide the assistance and services guaranteed by those rights” [1].

To respond to the challenges posed by the numerous and cited reports on human rights violations in mental health, the WHO (World Health Organization) launched the QualityRights project [20,21]. The project proposes an integrated approach, both top-down (to modify policies, and support and create user associations) and bottom-up (to improve the skills of mental health professionals and users’ awareness of their rights) [20].

Particularly, the WHO’s QualityRights toolkit offers practical guidance for assessing and improving human rights adherence in mental health facilities and is proving to be an essential resource for ensuring quality in these services [22]. Identifying areas for quality improvement through promoting human rights, facilitating recovery, and ensuring they reach appropriate standards, the initiative seeks to transform mental health care [20,23,24]. There is a growing expectation that mental health professionals will exhibit an increasing sensitivity to this matter, mirroring the expanding discourse both within the scientific community [25] and that these actions can translate, in countries adhering to these initiatives [26], into substantially shaping policies to improve mental health care quality. 

The occurrence of rights violations in mental health care services significantly contributes to organizational well-being. Indeed, the correlation between the adherence to users’ rights and service quality is evolving into a veritable benchmark of quality standards [27,28,29]. In an Italian study, the perception of the respect for human rights for users and staff by mental health professionals was found to strongly correlate to job satisfaction and individual well-being at work [30], even when the perception of users, while recognizing an excellent level of adherence to the standards of the respect for human rights, express a need for more resources and services [31]. It is not surprising that these dimensions of organizational well-being and satisfaction are interlinked; however, their relationship with psychosomatic health should nonetheless be considered. The research found a negative correlation between job satisfaction and symptoms such as headaches, concentration difficulties, anxiety, fatigue, and depression, underscoring the importance of recognizing the strengths and weaknesses of the service for organizational development [32,33]. 

In Latin America, mental health service reforms over the past two decades show limited progress, highlighting barriers like insufficient funding and a lack of consensus relevant for studying the staff perception of human rights and workplace well-being [34,35]. 

The primary objective of this study is to assess whether there are significant differences in the perception of human rights’ respect for users and staff, as well as organizational and job satisfaction among mental health professionals in three South American countries administering the well-being at work and respect for human rights (WWRR) questionnaire, which has already been utilized in other contexts.

It will also be speculated how the different models of health care policy and the cultural professional framework may influence the perception of the respect for staff and patients’ rights and well-being at work among the mental health workers. The findings will provide an insight into the perceived quality of mental health services, particularly regarding users’ and staff rights in Latin American counties [36].

## 2. Materials and Methods

### 2.1. Design 

This is an observational cross-sectional study conducted across seven distinct mental health services in three countries of South America (Argentina, Colombia, and Peru). Data collection began in October 2018 and concluded in July 2019. Participation was requested from several Latin American countries for this study; however, only these three countries successfully obtained the necessary authorization from their respective ethics committees.

### 2.2. Settings and Enrolment 

A convenience sampling method was used, based on participant availability and willingness to be enrolled. The inclusion criterion was working in private and public mental health services. Mental health service workers have been included from three different countries in South America: Argentina, Colombia, and Peru. The survey questionnaires were administered both online and using paper-based methods. All the health workers involved worked in mental health services, in roles such as nurses, general doctors, psychiatrists, rehabilitators, and psychologists, as well as administrative staff. In Peru and Argentina, the questionnaire was sent online through worker associations. In Colombia, a paper questionnaire was administered individually to 135 healthcare workers. All the participants provided written informed consent.

#### 2.2.1. Argentina 

The study involved personnel from community mental health services and hospital emergency departments in the province of Buenos Aires, specifically within the metropolis of La Plata, collaborated through a consortium of mental health experts.

#### 2.2.2. Colombia 

The research was performed in two public and private mental health services in Valledupar, in the Caribbean region. Professionals working in clinics, ambulatory care centers, and public health institutions, providing mental health and psychiatric care, were recruited.

#### 2.2.3. Peru

Mental health workers in psychiatric units and mental health centers for both outpatients and inpatients were selected across the nation, through the Consortium of Mental Health Experts. 

### 2.3. Measurement Tool

Each selected participant in the study completed a socio-demographic information form and filled out the validated instrument well-being at work and respect for human rights (WWRR) [37,38], which consists of seven items. The items are about staff’s satisfaction with job; staff’s satisfaction with organization; respect of staff’s human rights; respect of users’ human rights; staff’s satisfaction with resources for care; users’ satisfaction with care; and one last question about specific needs for professionals. 

The main factors of the investigation consist of the first six elements (well-being at work and respect for human rights) and are assessed through the following questions:

1. How much are you satisfied with your job?
2. How much do you think the users of your service ward are satisfied?
3. How much are you satisfied with the organizational aspect of your work/how your work is organized?
4. How much do you think the human rights of the users of your service/ward are respected?
5. How much do you think the human rights of your staff are respected?
6. How do you evaluate the current state of care in mental health in your service/ward, with reference to resources?

Item 7 essentially serves an informative purpose, and primarily aims to collect perceptions regarding the demand for specific human resources within the service. 

The questionnaire was also validated in Spanish [37].

### 2.4. Ethics 

The research protocol was approved by the Ethical Committee of the University Hospital of Cagliari, Italy, protocol number PG/2018/7337, and was conducted following the guidelines of the 1995 Declaration of Helsinki and its subsequent amendments [39]. All the participants provided written informed consent.

### 2.5. Statistical Analysis

The Statistical Package for Social Sciences (SPSS) version 20 was utilized for coding and analyzing all data. All tests conducted were two-tailed, with a significance level set at *p* < 0.05.

Continuous variables were presented as means with standard deviations, while categorical variables were described using counts and percentages. This approach provides a clear overview of the data’s central tendency and distribution.

For categorical data analysis, the Chi-square test was applied, with Yates’ correction being used when necessary [40]. This ensures accurate testing for independence between categorical variables.

The reliability of the questionnaire was evaluated using Cronbach’s alpha. A value of 0.70 or above is deemed satisfactory for group comparisons [41], ensuring that the questionnaire is consistent in measuring what it is intended to.

For comparing the participant groups by country, one-way analysis of variance (ANOVA) was employed. Eta-squared (η^2^) was used to measure the effect size within the ANOVA, where values around 0.01, 0.06, and 0.14 represent small, medium, and large effect sizes, respectively [42,43]. This allows for a nuanced understanding of the differences between groups.

## 3. Results

As can be seen from Table 1, the overall sample is made up of *n* = 310 mental health professionals. A higher percentage of females is noticeable, with Peru being the only exception (Table 1). The sample from Argentina is made up of workers younger than those from Colombia and Peru (20–29 years 30.9% Argentina vs. 19.8% Colombia, 16.2% Peru, *p* < 0.0001). This difference reaches a statistical significance (Table 1). 

Table 2 shows the differences between countries with respect to all the dimensions investigated by the survey through effect sizes, using eta-squared (Table 2). The three countries showed statistically significant differences with regards to some items investigated by the instrument. Participants from Colombia reflect a lower staff satisfaction with organization compared with the participants from Peru and Argentina (η^2^ = 0.113).

In comparison with Peru and Argentina, participants from Colombia also exhibit a significantly lower level of staff satisfaction with resources for care (η^2^ = 0.166). Although statistically significant differences were observed in these two dimensions, they were characterized by small effect sizes (Table 2).

In general, all participants from the three countries showed no significant differences in the other dimensions: staff’s satisfaction with job; respect of staff’s human rights; respect of users’ human rights; and users’ satisfaction with care. Approximately, the values of these dimensions tend to be medium–high for the three countries surveyed. However, Argentina shows a lower score in the respect of user’s human rights dimension, but it does not reach a statistical significance. 

**Figure 1:**
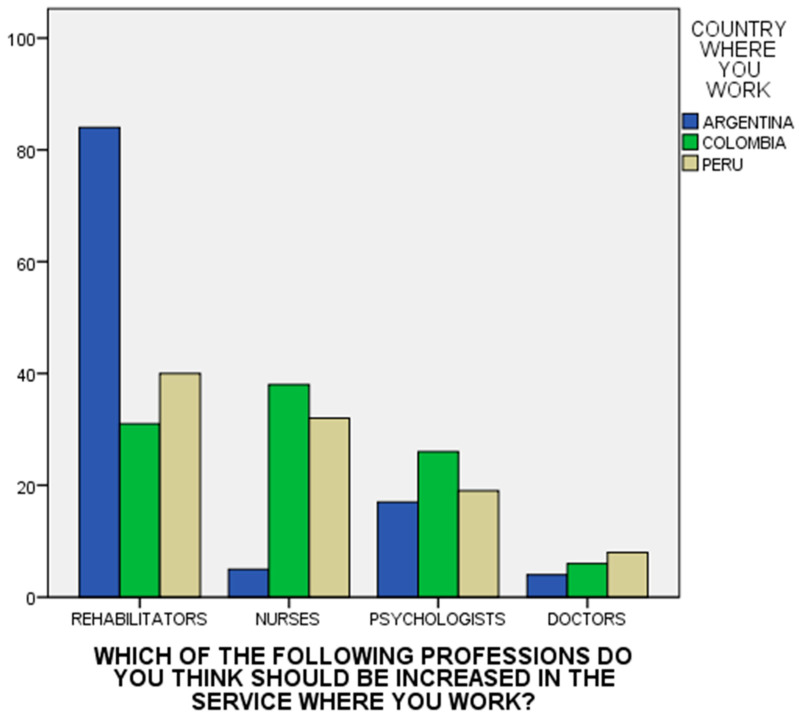
Distribution of scores on interventions required to improve resources by country.

Regarding the seventh question, investigating which professionals the individual thinks should be increased in the service where they work, the participants suggested increasing the number of nurses and rehabilitators (*χ*^2^ = 57.042; df = 6; *p* < 0.0001) (see Figure 1). The assessment tool was reliable when measured with the Cronbach’s alpha: Argentina = 0.759 (95% CI: 0.68–0.82); Colombia= 0.748 (0.66–0.81); and Peru = 0.73 (0.64–0.80).

## 4. Discussion

This study offers a comparative view of satisfaction and perceptions of the respect for human rights and organizational well-being among mental health professionals in Argentina, Colombia, and Peru. 

A salient feature across the sample is the dominance of females among the participants. This is consistent with previous studies indicating the predominance of females in healthcare professions, especially in the nursing and caregiving sectors both globally and in South America [44]. 

Well-being at work and respect for human rights (WWRR) reveals significant differences among countries. In particular, Colombian participants reported lower scores in the dimensions of satisfaction with the organization and satisfaction with the resources for care, compared to Peru and Argentina. This aligns with the findings by Orozco et al. [45] and Campo-Arias et al. [46,47], which suggest that inadequate resources and organizational inefficiencies can significantly hinder the provision of optimal mental health services. In contrast, Argentine participants showed a higher level in the dimension of staff satisfaction with resources for care and in the dimension of users’ satisfaction with care. This could point to a better-resourced mental health infrastructure in Argentina, which may be due to the creation of new laws that regulate mental health and promote investment by the health system of that country [48,49]. This nation has reformed its mental health system, transitioning to community-centered care with a strong emphasis on human rights [49]. Despite challenges in nationwide execution due to regional variations, significant strides have been made in service accessibility, workforce training, and policy implementation. The reforms in Buenos Aires exemplify this rapid transformation [50] and the increasing consensus garnered by the movement of mental health users who defend their rights [51,52,53]. A similar situation is observed when comparing the results of the same study carried out in the Mediterranean area [36]. The perception of the resources available in the Italian mental health services is much more consistent and diversified compared to the other three countries that were part of the study (Macedonia, Tunisia, and Palestine). In this case as well, the characteristics of the social and legal context likely have an impact on the perceptions. Community psychiatry, exemplified by the deinstitutionalization movement spurred by the Basaglia Law in Italy [54], represents a transformative approach in mental health care. This model, which Argentina is also attempting to implement [55], advocates for shifting away from centralized psychiatric hospitals, prioritizing community-based care and the integration of individuals with mental health conditions into society. This paradigm shift can foster a perception among mental health professionals of more equitable resource allocation compared to nations where mental health care remains hospital-centric [56]. 

As concerns the dimensions of respect for users’ human rights and respect for staff’s human rights, Colombia obtained the highest scores. This could be explained by the increasing focus on human rights in Colombia’s public policies. Starting with the enactment of Law 1616 in 2013 [57,58], which prioritizes mental health in Colombia and encompasses various mental health care modalities and services, followed by the Statutory Law of Health (Law 1751 of 2015) considering social determinants as integral to understanding health and disease, recognizing it as a fundamental right [59]. Additionally, the Policies of Mental Health and Prevention, along with the Attention of the Consumption of Psychoactive Substances (2018–2019), emphasize a commitment to human rights, primary health care, gender considerations, and social determinants [60]. 

Regarding the interpretation of low scores in the perception of resources and organizational well-being, it is not unlikely that these may also stem from a renewed emphasis on service quality, prompted by the shift in perspective brought about by a focus on human rights. Nevertheless, considering the overall historical trajectory of Latin American countries, investments in mental health have been comparatively lower than in other subregions and countries with similar income levels [61]. 

For a long time, there has been a lack of strong political will to implement reforms, minimal allocations of healthcare resources for mental health, the absence of legislation to safeguard the human rights of individuals with mental disorders, and almost a complete absence of anti-stigma and mental health literacy policies [13]. 

It is equally evident, however, that the transition from the hospital-centric psychiatric model to the community-based model is a process that requires time [62]. However, a trend towards incorporating the standards of the Convention on the Rights of Persons with Disabilities (CRPD) into mental health regulation was discernible in the three jurisdictions [63]. This encompassed areas such as decision-making support, implementing advance directives, and, overall, safeguarding an individual’s will and preferences during treatment. With regards to involuntary hospitalization, instances of hospitalization and emergency psychiatric mandatory treatment are maintained. In fact, Argentina has retained hospitalization and emergency psychiatric treatment without significant alterations in coercive measures, while Peru has shown promising progress in eliminating them [64] via training and awareness programs in mental health and community engagement [65,66]. Prior to the introduction of the proposed legislation in Peru, a 2008 study by Pedersen et al. assessed the high prevalence and distribution of mental health problems, exploring the connection with the social context and considering the implications for both mental health interventions and human rights [66]. The situation in Colombia is still evolving, with the reform of legal capacity having the effect of revoking specific regulations related to mental health admissions [63]. 

Surprisingly, in Argentina, Colombia, and Peru, relatively high scores are evident in the dimension “staff satisfaction with job” despite the low economic investment, as reported by the WHO in the Mental Health ATLAS 2020. The Americas region invests minimally in mental health resources, contributing only 1.8% compared to the global average of 2.13% [67]. This observation contrasts with previous research from various cultural contexts and health systems, such as those in Ethiopia, China, and Italy. These studies demonstrate how the lack of resources can indirectly influence the job satisfaction of health professionals [15,68,69].

Ultimately, the study participants emphasized the importance of integrating a larger number of rehabilitation specialists and nursing personnel into these services, highlighting the indispensable role these experts play in comprehensive mental health care. This recommendation aligns with worldwide initiatives aimed at fortifying community-based rehabilitation services and expanding the nursing workforce to address the growing requirements in mental healthcare [70,71,72]. It is important to note that many of the respondents are from the nursing profession, and this could condition the results. In the case of Argentina, the main responses were from nurses and psychologists. This country has a history of vision of the social importance of the role of psychologists and, in fact, has the largest number of psychologists per capita in the world [67,73]. Colombia and Peru, despite needing many rehabilitators, also feel a lack of doctors and nurses; particularly in Colombia, they would like more nurses than rehabilitators. This suggests that there is still a trend toward a hospital-centric model. Comparing these results with the previous study in the Mediterranean [36], something similar happens in Italy, where a demand for more doctors and psychologists suggests, with a smaller proportion of rehabilitators, the presence of a hospital-oriented vision of mental health care services and less comprehensive social and labor inclusion [74]. 

Despite improvements in compliance with coercion-free medical standards and greater attention to mental health, these countries still face challenges derived from underdevelopment and social problems. Addressing these challenges, along with efforts to destigmatize mental health and also ensuring adequate quality standards in terms of organizational well-being, remains a key priority for the region [75]. 

Further policy reforms and resource allocation tailored to mental health professionals in Argentina, Colombia, and Peru are needed. Key focuses include improving workplace satisfaction and human rights respect, given the close link they seem to have with the dimension of organizational well-being, and addressing the disparities in resources and organizational support. The study highlights the need for more thoroughly adapted mental health care models to recovery-oriented, community-based approaches, which are inclusive and free from coercion. These actions aim to enhance the quality of mental health services and the well-being of both professionals and patients in these countries.

## 5. Limitations

While our study provides valuable and noteworthy insights into the experiences and perceptions of mental health professionals, it is important to acknowledge certain limitations that may have influenced the results. One such limitation is the self-report nature of the questionnaire used in the study, which could potentially introduce response biases, due to the subjective nature of the responses. Participants might have responded in a manner that they deemed socially acceptable or favorable, rather than providing responses that accurately reflect their true experiences and perceptions. 

Furthermore, the participants in this study were not selected through a random sampling method, but rather through voluntary participation. This means that the questionnaire was likely filled out by individuals who were the most interested and motivated to participate, which could potentially skew the results. It is possible that those who chose to participate have different experiences or perceptions compared to those who did not participate, and this should be taken into consideration when interpreting the findings.

Furthermore, the response rate is not presented, as it was not possible, with the exception of a few associations, to ascertain the total number of online questionnaire submissions and the extent of the initiative’s dissemination among all potential participants. 

It should be emphasized that the selection of mental health centers that could participate was also affected by a selection bias too. Despite the request being sent to universities in various South American countries, only some responded positively to the initiative through associations. Therefore, the sample recruited is not representative of the population of mental health professionals in the countries that participated. Moreover, the survey questionnaires were administered through both online and paper-based methods, which could have introduced variability in the responses. Different modes of administration might have influenced the way participants interpreted and responded to the questions, potentially affecting the consistency and reliability of the data.

Individual and country-specific factors, such as policy changes, funding patterns, and socio-cultural attitudes deserve to be explored through an analysis that evaluates the actual relationship between them, providing a more nuanced understanding of the observed differences between the experiences and perceptions of mental health professionals in the three Latin American countries studied. A more detailed analysis, indeed, could reveal how these factors interact and contribute to the overall experiences of mental health professionals and their satisfaction and perception beyond all speculation.

Finally, it is important to note that the majority of respondents in this study are nurses and psychologists. This demographic distribution could have conditioned the responses to the questions, as these professionals might have unique perspectives and experiences that differ from those of other mental health professionals.

## 6. Conclusions 

This study offers an initial and preliminary overview of the experiences and perceptions of mental health professionals in three South American countries regarding respect for human rights and organizational satisfaction within their work contexts. There is a need to extend research to other Latin American nations among mental health practitioners to highlight the level of perception in adherence to CRPD standards, aiming for greater homogeneity and an awareness of these issues in the future. Furthermore, the findings underscore the need for further large-scale investigations into the role of political and practical contexts in ensuring that mental health services adhere to international quality standards. Such standards closely link respect for human rights with well-being at work, ultimately improving the experiences of users and the quality of care they receive. This harmonization not only enhances professional environments, but also directly contributes to the elevation of patient care standards across the board.

## Figures and Tables

**Table 1:** General characteristics of the participants.

-	Argentina	Colombia	Peru	Chi-Square
-	*n* = 110	*n* = 101	*n* = 99	
Male	16 (14.5%)	34 (33.7%)	40 (40.4%)	*χ*^2^ = 18.47; df = 2; *p* = 0.000
Female	94 (85.5%)	67 (66.3%)	59 (59.6%)	
<20 Years old	0 (0.00%)	0 (0.00%)	1 (1.00%)	*χ*^2^ = 20.09; df = 10; *p* = 0.028
20–29 years old	34 (30.9%)	20 (19.8%)	16 (16.2%)	
30–39 years old	36 (32.7%)	42 (41.6%)	42 (42.4%)	
40–49 years old	20 (18.2%)	16 (15.8%)	24 (24.2%)	
50–59 years old	18 (16.4%)	12 (11.9%)	11 (11.1%)	
>60 years old	2 (1.8%)	11 (10.9%)	5 (5.1%)	

**Table 2:** Comparison by country of scores based on the quality rights assessment tool.

All Data: Mean (Standard Deviation)	Argentina	Colombia	Peru	ANOVA	Effect Size
-	*n* = 110	*n* = 101	*n* = 99	-	-
Staff’s satisfaction with job	4.48 SD 1.406	4.48 SD 1.171	4.84 SD 0.866	F(3, 142) = 8.71, *p* = 0.045	η^2^ = 0.029
Staff’s satisfaction with organization	4.66 SD 1.422	4.07 SD 1.283	4.72 SD 0.770	F(2, 970) = 5.828, *p* = 0.053	η^2^ = 0.113
Respect of staff’s human rights	4.35 SD 1.59	4.96 SD 1.174	4.67 SD 1.040	F(9, 109) = 26.298, *p* = 0.00	η^2^ = 0.080
Respect of users’ human rights	4.88 SD 1.262	4.91 SD 1.167	4.74 SD 0.864	F(0, 695) = 1.734, *p* = 0.500	η^2^ = 0.004
Staff’s satisfaction with resources for care	2.93 SD 0.955	3.74 SD 1.083	3.19 SD 0.853	F(5, 884) = 19.960, *p* = 0.003	η^2^ = 0.166
Users’ satisfaction with care	5.00 SD 1.157	4.67 SD 1.078	4.9 SD 0.631	F(19, 216) = 36.063, *p* = 0.000	η^2^ = 0.019

SD: standard deviation F × *N* = η^2^ × *p*.

## Data Availability

The data collected for this study were entirely published.

## References

[1] Convention on the Rights of Persons with Disabilities (CRPD)|Division for Inclusive Social Development (DISD). https://social.desa.un.org/issues/disability/crpd/convention-on-the-rights-of-persons-with-disabilities-crpd.

[2] McCusker P., Gillespie L., Davidson G., Vicary S., Stone K. (2023). The United Nations Convention on the Rights of Persons with Disabilities and Social Work: Evidence for Impact?. Int. J. Environ. Res. Public Health.

[3] I Diritti Umani Nel 2022: L’analisi Di Amnesty International. https://www.amnesty.it/rapporti-annuali/rapporto-2022-2023/analisi-globale/.

[4] Mji G., Rhoda A., Statham S., Joseph C. (2017). A Protocol for the Methodological Steps Used to Evaluate the Alignment of Rehabilitation Services in the Western Cape, South Africa with the National Rehabilitation Policy. BMC Health Serv. Res..

[5] Jesus T.S., Landry M.D., Dussault G., Fronteira I. (2017). Human Resources for Health (and Rehabilitation): Six Rehab-Workforce Challenges for the Century. Hum. Resour. Health.

[6] Mahdanian A.A., Laporta M., Drew Bold N., Funk M., Puras D. (2023). Human Rights in Mental Healthcare; A Review of Current Global Situation. Int. Rev. Psychiatry.

[7] (2017). Report of the Special Rapporteur on the Right of Everyone to the Enjoyment of the Highest Attainable Standard of Physical and Mental Health. https://reliefweb.int/report/world/report-special-rapporteur-right-everyone-enjoyment-highest-attainable-standard-0.

[8] 49th Regular Session of the Human Rights Council (28 February–1 April 2022). https://www.ohchr.org/en/hr-bodies/hrc/regular-sessions/session49/regular-session.

[9] (2004). The Right of Everyone to the Enjoyment of the Highest Attainable Standard of Physical and Mental Health: Report of the Special Rapporteur, Paul Hunt. https://digitallibrary.un.org/record/517605?ln=en.

[10] Wigand M.E., Orzechowski M., Nowak M., Becker T., Steger F. (2021). Schizophrenia, Human Rights and Access to Health Care: A Systematic Search and Review of Judgements by the European Court of Human Rights. Int. J. Soc. Psychiatry.

[11] Funk M., Drew N. (2019). Practical Strategies to End Coercive Practices in Mental Health Services. World Psychiatry.

[12] Vigo D., Thornicroft G., Atun R. (2016). Estimating the True Global Burden of Mental Illness. Lancet Psychiatry.

[13] Javed A., Lee C., Zakaria H., Buenaventura R.D., Cetkovich-Bakmas M., Duailibi K., Ng B., Ramy H., Saha G., Arifeen S. (2021). Reducing the Stigma of Mental Health Disorders with a Focus on Low- and Middle-Income Countries. Asian J. Psychiatry.

[14] Perlin M.L., Perlin M.L. (2011). International Human Rights Law in Perspective: Legal Issues and Social Constructs. International Human Rights and Mental Disability Law: When the Silenced Are Heard.

[15] Carta M.G., Angermeyer M.C., Sancassiani F., Tuligi F., Pirastu R., Pisano A., Pintus E., Mellino G., Pintus M., Pisanu E. (2013). A Follow-up on Patients with Severe Mental Disorders in Sardinia after Two Changes in Regional Policies: Poor Resources Still Correlate with Poor Outcomes. BMC Psychiatry.

[16] Nankivell J., Platania-Phung C., Happell B., Scott D. (2013). Access to Physical Health Care for People with Serious Mental Illness: A Nursing Perspective and a Human Rights Perspective-Common Ground?. Issues Ment. Health Nurs..

[17] Ventura C.A.A., Austin W., Carrara B.S., de Brito E.S. (2021). Nursing Care in Mental Health: Human Rights and Ethical Issues. Nurs. Eth..

[18] Pathare S., Funk M., Bold N.D., Chauhan A., Kalha J., Krishnamoorthy S., Sapag J.C., Bobbili S.J., Kawade R., Shah S. (2021). Systematic Evaluation of the QualityRights Programme in Public Mental Health Facilities in Gujarat, India. Br. J. Psychiatry.

[19] Chatterjee S., Pathare S., Funk M., Drew-Bold N., Das P., Chauhan A., Kalha J., Krishnamoorthy S., Sapag J.C., Bobbili S.J. (2023). Cost of Implementing the QualityRights Programme in Public Hospitals in Gujarat Providing Mental Healthcare. Indian J. Med. Res..

[20] Funk M., Drew N. (2017). WHO QualityRights: Transforming Mental Health Services. Lancet Psychiatry.

[21] https://qualityrights.org/wp-content/uploads/QRFlyer-2022-for-Web.pdf.

[22] https://www.who.int/publications/i/item/who-qualityrights-guidance-and-training-tools.

[23] Harden B., Gyimah L., Funk M., Drew-Bold N., Orrell M., Moro M.F., Cole C., Ohene S.-A., Baingana F., Amissah C. (2023). Attitudes towards Persons with Mental Health Conditions and Psychosocial Disabilities as Rights Holders in Ghana: A World Health Organization Study. BMC Psychiatry.

[24] Carta M.G., Sancassiani F., Melis P., Aviles-Gonzales C.I., Urban A., Minerba L., D’Oca S., Atzeni M., Velluzzi F., Ferreli C. (2022). The Perception of Professionals and Users of the Quality of Care and Respect for Human Rights in Four Outpatient Care Facilities of an Italian Hospital during the COVID-19 Pandemic. J. Public Health Res..

[25] Moro M.F., Pathare S., Zinkler M., Osei A., Puras D., Paccial R.C., Carta M.G. (2021). The WHO QualityRights Initiative: Building Partnerships among Psychiatrists, People with Lived Experience and Other Key Stakeholders to Improve the Quality of Mental Healthcare. Br. J. Psychiatry J. Ment. Sci..

[26] Moro M.F., Kola L., Fadahunsi O., Jah E.M., Kofie H., Samba D., Thomas S., Drew N., Nwefoh E., Pathare S. (2022). Quality of Care and Respect of Human Rights in Mental Health Services in Four West African Countries: Collaboration between the Mental Health Leadership and Advocacy Programme and the World Health Organization QualityRights Initiative—ADDENDUM. BJPsych Open.

[27] QR E-Training. https://www.who.int/teams/mental-health-and-substance-use/policy-law-rights/qr-e-training.

[28] WHO QualityRights Tool Kit. https://www.who.int/publications-detail-redirect/9789241548410.

[29] Nomidou A. (2013). Standards in Mental Health Facilities—An in Depth Case Study in Greece Using the WHO QualityRights Tool. J. Public Ment. Health.

[30] Carta M.G., Moro M.F., Sancassiani F., Ganassi R., Melis P., Perra A., D’Oca S., Atzeni M., Velluzzi F., Ferreli C. (2022). Respect for Service Users’ Human Rights, Job Satisfaction, and Wellbeing Are Higher in Mental Health Workers than in Other Health Workers: A Study in Italy at Time of the Covid Pandemic. J. Public Health Res..

[31] Cossu G., Zreik T., Ciccu S., Guttman M.E., Sancassiani F., Melis P., Angermeyer M., Carta M.G. (2023). Respects of Human Rights and Perception of Quality of Care, the Users’ Point of View Comparing Mental Health and Other Health Facilities in a Region of Italy. Int. Rev. Psychiatry.

[32] Angermeyer M.C., Schomerus G., Carta M.G., Moro M.F., Toumi M., Millier A., Holzinger A. (2013). Burnout: Ein deutsches Phänomen?. Psychiatr. Prax..

[33] Carta M.G., Ghacem R., Milka M., Moula O., Staali N., Uali U., Boukhari G., Mannu M., Refrafi R., Yaakoubi S. (2021). Implementing WHO-Quality Rights Project in Tunisia: Results of an Intervention at Razi Hospital. Clin. Pract. Epidemiol. Ment. Health.

[34] Caldas de Almeida J.M. (2013). Mental Health Services Development in Latin America and the Caribbean: Achievements, Barriers and Facilitating Factors. Int. Health.

[35] (2013). Robles, Enrique Camarena, y Edgard G. Belfort. The Reality of the Management of Mental Health Services in Latin America, Reflexions from the Perspective of a Vision of Strategic Planning. Int. Rev. Psychiatry.

[36] Zgueb Y., Preti A., Perra A., El-Astal S., Gonzalez C.I.A., Piras M., Testa G., Kirolov I., Tamburini G., Ouali U. (2020). Staff Perception of Respect for Human Rights of Users and Organizational Well-Being: A Study in Four Different Countries of the Mediterranean Area. Clin. Pract. Epidemiol. Ment. Health.

[37] Aviles Gonzalez C.I., Galletta M., Cerchiaro Fernandez D.M., Guerra Muñoz M.E., Abarca Arias Y.M., Brasesco M.V., Atzeni M., Romano F., Primavera D. (2023). Respect for Human Rights as a Component of Organisational Well-Being: Factor Structure Analysis in Three Countries of Latin America. Int. Rev. Psychiatry.

[38] Husky M., Zgueb Y., Ouali U., Gonzalez C.I.A., Piras M., Testa G., Maleci A., Mulas A., Montisci A., Nujedat S. (2020). Principal Component Analysis of the Well-Being at Work and Respect for Human Rights Questionnaire (WWRRR) in the Mediterranean Region. Clin. Pract. Epidemiol. Ment. Health.

[39] (2013). World Medical Association Declaration of Helsinki: Ethical Principles for Medical Research Involving Human Subjects. JAMA.

[40] Giannini E.H., Cassidy J.T., Petty R.E., Laxer R.M., Lindsley C.B. (2005). CHAPTER 6—Design, Measurement, and Analysis of Clinical Investigations. Textbook of Pediatric Rheumatology.

[41] Kottner J., Streiner D.L. (2010). Internal Consistency and Cronbach’s α: A Comment on Beeckman et al. (2010). Int. J. Nurs. Stud..

[42] Ruxton G.D., Beauchamp G. (2008). Time for Some a Priori Thinking about Post Hoc Testing. Behav. Ecol..

[43] Zach What Is Eta Squared? (Definition & Example). https://www.statology.org/eta-squared/.

[44] Lotta G., Fernandez M., Pimenta D., Wenham C. (2021). Gender, Race, and Health Workers in the COVID-19 Pandemic. Lancet.

[45] Orozco R., Vigo D., Benjet C., Borges G., Aguilar-Gaxiola S., Andrade L.H., Cia A., Hwang I., Kessler R.C., Piazza M. (2022). Barriers to Treatment for Mental Disorders in Six Countries of the Americas: A Regional Report from the World Mental Health Surveys. J. Affect. Disord..

[46] Cassiani S.H.D.B., Munar Jimenez E.F., Umpiérrez Ferreira A., Peduzzi M., Leija Hernández C. (2020). La situación de la enfermería en el mundo y la Región de las Américas en tiempos de la pandemia de COVID-19. Rev. Panam. Salud Pública.

[47] Campo-Arias A., Oviedo H.C., Herazo E. (2014). Estigma: Barrera de Acceso a Servicios En Salud Mental. Rev. Colomb. Psiquiatr..

[48] Campo-Arias A., Ceballos-Ospino G.A., Herazo E. (2020). Barriers to Access to Mental Health Services among Colombia Outpatients. Int. J. Soc. Psychiatry.

[49] Cohen H. (2022). La Ley de Salud Mental en Argentina [Mental health law in Argentina]. Medicina.

[50] Moldavsky D., Cohen H. (2013). The New Mental Health Law in Argentina. Int. Psychiatry.

[51] Corin M. (2013). Community mental health networks and doors at a Health and Community Center (CeSAC No 24) in the south of the Autonomous City of Buenos Aires (CABA). Vertex.

[52] Barcala A., Faraone S. (2023). Advancements in Mental Health Reform in Argentina: Towards Comprehensive and Human Rights-Respecting Care. Lancet Reg. Health Am..

[53] Ardila-Gómez S., Agrest M., Fernández M.A., Rosales M., López L., Velzi Díaz A.R., Vivas S.J., Ares Lavalle G., Basz E., Scorza P. (2019). The Mental Health Users’ Movement in Argentina from the Perspective of Latin American Collective Health. Glob. Public Health.

[54] Istituto Poligrafico e Zecca dello Stato LEGGE 13 Maggio 1978, n. 180—Normattiva. https://www.normattiva.it/uri-res/N2Ls?urn:nir:stato:legge:1978-05-13;180!vig=.

[55] Barcala A., Faraone S. (2023). Mental Health Reforms in Buenos Aires, Argentina. Lancet Psychiatry.

[56] Carta M.G., Angermeyer M.C., Holzinger A. (2020). Mental Health Care in Italy: Basaglia’s Ashes in the Wind of the Crisis of the Last Decade. Int. J. Soc. Psychiatry.

[57] Ley 1616 de 2013—Salud Mental. https://www.asivamosensalud.org/politicas-publicas/normatividad-leyes/salud-publica/ley-1616-de-2013-salud-mental.

[58] Santamaría-García H. (2023). Mental Health in the Current Social Scenario in Colombia. Rev. Colomb. Psiquiatr. Engl. Ed..

[59] Leyes Desde 1992—Vigencia Expresa y Control de Constitucionalidad [LEY_1751_2015]. http://www.secretariasenado.gov.co/senado/basedoc/ley_1751_2015.html.

[60] Agudelo-Hernández F., Rojas-Andrade R. (2023). Mental Health Services in Colombia: A National Implementation Study. Int. J. Soc. Determ. Health Health Serv..

[61] Minoletti A., Galea S., Susser E. (2012). Community Mental Health Services in Latin America for People with Severe Mental Disorders. Public Health Rev..

[62] Kohn R., Ali A.A., Puac-Polanco V., Figueroa C., López-Soto V., Morgan K., Saldivia S., Vicente B. (2018). Mental Health in the Americas: An Overview of the Treatment Gap. Rev. Panam. Salud Pública.

[63] Marshall P. (2023). Argentina, Chile, Colombia, and Peru: The Relationship of Mental Health Law and Legal Capacity. Routledge Handbook of Mental Health Law.

[64] Arriola-Vigo J.A., Stovall J.G., Moon T.D., Audet C.M., Diez-Canseco F. (2019). Perceptions of Community Involvement in the Peruvian Mental Health Reform Process Among Clinicians and Policy-Makers: A Qualitative Study. Int. J. Health Policy Manag..

[65] Scorza P., Cutipe Y., Mendoza M., Arellano C., Galea J.T., Wainberg M.L. (2019). Lessons from Rural Peru in Integrating Mental Health into Primary Care. Psychiatr. Serv..

[66] Pedersen D., Tremblay J., Errázuriz C., Gamarra J. (2008). The Sequelae of Political Violence: Assessing Trauma, Suffering and Dislocation in the Peruvian Highlands. Soc. Sci. Med..

[67] Mental Health ATLAS 2020. https://www.who.int/publications-detail-redirect/9789240036703.

[68] Huang X., Chen H., Gao Y., Wu J., Ni Z., Wang X., Sun T. (2022). Career Calling as the Mediator and Moderator of Job Demands and Job Resources for Job Satisfaction in Health Workers: A Cross-Sectional Study. Front. Psychol..

[69] Hotchkiss D.R., Banteyerga H., Tharaney M. (2015). Job Satisfaction and Motivation among Public Sector Health Workers: Evidence from Ethiopia. Hum. Resour. Health.

[70] Adams R., Ryan T., Wood E. (2021). Understanding the Factors That Affect Retention within the Mental Health Nursing Workforce: A Systematic Review and Thematic Synthesis. Int. J. Ment. Health Nurs..

[71] Delaney K.R., Vanderhoef D. (2019). The Psychiatric Mental Health Advanced Practice Registered Nurse Workforce: Charting the Future. J. Am. Psychiatr. Nurses Assoc..

[72] Dalton-Locke C., Marston L., McPherson P., Killaspy H. (2021). The Effectiveness of Mental Health Rehabilitation Services: A Systematic Review and Narrative Synthesis. Front. Psychiatry.

[73] Onofrio G.B., Kitroser N. (2021). Psychodynamic Psychiatry in Argentina at a Crossroads. Psychodyn. Psychiatry.

[74] Barlati S., Stefana A., Bartoli F., Bianconi G., Bulgari V., Candini V., Carrà G., Cavalera C., Clerici M., Cricelli M. (2019). Violence Risk and Mental Disorders (VIORMED-2): A Prospective Multicenter Study in Italy. PLoS ONE.

[75] Cía A.H., Rojas R.C., Adad M.A. (2010). Current Clinical Advances and Future Perspectives in the Psychiatry/Mental Health Field of Latin America. Int. Rev. Psychiatry.

